# Laboratory professionals’ attitudes towards ISO 15189:2012 accreditation: an anonymous survey of three Croatian accredited medical laboratories

**DOI:** 10.11613/BM.2021.020712

**Published:** 2021-06-15

**Authors:** Ivana Lapić, Dunja Rogić, Matea Ivić, Marina Tomičević, Mirjana Mariana Kardum Paro, Lovorka Đerek, Ines Alpeza Viman

**Affiliations:** 1 Department of Laboratory Diagnostics, University Hospital Center Zagreb, Zagreb, Croatia; 2 Department of Medical Biochemistry and Laboratory Medicine, University Hospital Merkur, Zagreb, Croatia; 3 Clinical Department for Laboratory Diagnostics, University Hospital Dubrava, Zagreb, Croatia

**Keywords:** accreditation, quality assurance, ISO 15189:2012, survey, satisfaction

## Abstract

**Introduction:**

Effective implementation and continual compliance with ISO 15189:2012 require ongoing commitment and active involvement of laboratory staff. Our aim was to assess attitudes regarding accreditation implementation by conducting a survey in three Croatian accredited medical laboratories.

**Materials and methods:**

An anonymous survey consisting of 34 questions was distributed either electronically or in a paper form a week prior to scheduled annual audits. Distributions of answers regarding age, work experience, laboratory workplace, and education level and according to the respective laboratory were compared.

**Results:**

The overall response rate was 76% (225/297). Preference towards working in an accredited laboratory and a positive attitude were revealed by 70% and 56% participants, respectively, with better process documentation as the main advantage. Only 14% of responders considered themselves completely familiar with ISO 15189:2012. Total of 68% of responders felt that accreditation increases the usual workload, with excessive paperwork as the main contributor. Half of the responders declared partial agreement that accreditation requirements and expectations were clearly explained and claimed that their suggestions were taken into account only occasionally, which was especially emphasized by technical staff. The vast majority (89%) completely follow the prescribed protocols. Only 27% consider turnaround time monitoring useful. Competence assessment is considered efficient by 41% of responders. The majority (73%) prefer an online audit in times of COVID-19.

**Conclusions:**

Despite an overall positive attitude towards accreditation, further efforts are needed in providing better education about ISO 15189:2012 for technical staff and modifying formats of competence assessment, in order to achieve better adherence to ISO 15189:2012 requirements.

## Introduction

Accreditation of medical laboratories according to the International Organization for Standardization (ISO) 15189:2012 standard is a formal approval of competence given by a recognized authoritative body which confirms that the testing laboratory has proven all the technical competence as well as adequate quality of the total testing process (TTP) coupled with consistency of the management system which enables continuous delivery of valid and reliable examination results for the intended clinical use (*1*, *2*). The ISO 15189:2012 requirements address the need to define and document processes and procedures within the entire TTP, provide accuracy, safety and efficiency of laboratory services through rigorous quality assurance, ensure and maintain staff competence as well as promote continual improvement (*2*, *3*). By complying to ISO 15189:2012, laboratories demonstrate the ability to consistently provide high quality of service, and ultimately guarantee effective patient management in terms of improved patient safety and better clinical outcomes (*2*-*5*). However, implementation of accreditation standards in practice and day to day compliance with ISO 15189:2012 requirements are challenging and demanding, requiring great efforts and active involvement from all levels of laboratory staff in order to maintain full conformity to the specified objectives within time and costs constraints (*2*, *6*, *7*). While the myriad advantages of accreditation in terms of standardization and traceability of the TTP as well as improvement in quality of laboratory services and patient care are unquestionable and well-documented (*8*), adherence to such a standardized framework inevitably causes a shift in the usual laboratory workflow and implies additional workload. Nonetheless, published data shows that accreditation is well-accepted among laboratory staff, with satisfaction increasing over time (*9*).

ISO 15189:2012 provides a comprehensive guidance for establishment of a quality management system, but without defining how each specific clause should be addressed in practice. Therefore, practical implementation of ISO 15189:2012 requirements might vary between different laboratory settings (*10*, *11*). Laboratory staff competence and their understanding of the rationale beneath accreditation are crucial for effective compliance with ISO 15189:2012, while their feedback on the practical applicability of predefined protocols and working procedures should be inevitably taken into consideration in the process of quality system improvement (*2*, *10*).

Accreditation of medical laboratories in Croatia is carried out on a voluntary basis by the Croatian Accreditation Agency and so far only eight (six public and two private) out of 180 medical biochemistry laboratories have been accredited according to ISO 15189:2012, which is among the lowest rates of accredited medical laboratories compared to other European countries (*12*, *13*). Given the key role of laboratory staff in delivering accreditation requirements in routine practice (*2*), we conducted an anonymous survey encompassing staff across three accredited medical biochemistry laboratories, with all of them located within clinical hospitals. The aim of the study was to assess the attitudes of laboratory staff regarding implementation of various requirements of ISO 15189:2012 within their working environment, and in that way identify weak points, hence provide a basis for future improvements that could lead to better adherence to ISO 15189:2012. Additionally, the differences that might arise from variations in practical implementation of ISO 15189:2012 into routine practice between the included laboratories, as well as regarding the duration of accreditation status, were evaluated.

## Materials and methods

### Study design

The survey was conducted in October 2020 and included all laboratory professionals within the following three Croatian medical biochemistry laboratories accredited in compliance with ISO 15189:2012: Department of Laboratory Diagnostics of the University Hospital Center Zagreb, Department of Medical Biochemistry and Laboratory Medicine of the University Hospital (UH) Merkur and Clinical Department for Laboratory Diagnostics of the UH Dubrava. The laboratory of the UH Merkur has been accredited since 2007, while the other two laboratories since 2014.

The questionnaire included 34 questions (Q) divided into four sections: the first part included questions related to participants’ demographics (Q1-Q9), the second part comprised questions related to general attitude towards accreditation and familiarity with ISO 15189:2012 (Q10-Q12), the third part was focused on specific issues concerning implementation of accreditation requirements in routine practice (Q13-Q30) and the last four questions (Q31-Q34) dealt with accreditation audits. All questions were closed, single-choice, except questions 17 and 18 dealing with the opinion on the main advantages and disadvantages of accreditation. The latter questions had predefined options as well as an open field for possible additional answers and comments.

The survey was distributed either electronically using Google Forms application or in a paper form, depending on computer literacy of the participant. The survey was carried out independently during the one-week period before the annual accreditation audit scheduled for each respective laboratory. Because of the coronavirus disease 2019 (COVID-19) pandemic, at the time of conducting this survey it was still uncertain whether the accreditation audit would take place on-site or in a virtual form.

The survey was anonymous and participation in it was voluntary. For this type of study, ethical approval is not required while informed consent is implied, *i.e.* by accepting to participate, responders gave their informed consent for collection and dissemination of results.

### Data collection and analysis

Data was collected using the Google Forms platform and initial data analysis was performed by counting. For surveys completed in a paper form, responses were manually entered in Google Forms. Incomplete or vague responses were not taken into consideration and were defined as ‘no answer’. Results are presented as absolute numbers and percentages of the total responders’ number.

Moreover, to gain a more profound insight into attitudes towards accreditation, participants were divided into appropriate groups according to demographic data. Frequencies of answers were further compared according to age (≤ 40 and > 40 years), work experience (≤ 10 and > 10 years), duration of work at the current workplace (≤ 10 and > 10 years), level of education (technical staff including medical laboratory technicians who graduated from four-year secondary school only and bachelors of medical laboratory diagnostics holding a 3-year university degree *vs.* academic staff spanning from employees holding a master degree, with or without laboratory medicine specialization or a PhD degree) and laboratory workplace (routine laboratory including the preanalytical section, emergency laboratory and core laboratory *vs.* specialized laboratories).

Differences between the three included laboratories were also tested. Additionally, difference in distribution of answers between the one laboratory accredited for 14 years compared to the other two accredited for 7 years, was assessed. For questions 11, 12, 13, 14 and 32 the distribution of answers could not meet the requirements of the statistical test. Therefore, answers were merged into categories as follows: ‘positive’ *vs.* ‘neutral/negative’ (Q11), ‘Not at all/Moderately’ *vs.* ‘Very well/Completely’ (Q12), ‘Totally agree’ *vs.* ‘I partially agree/I do not agree’ (Q13 and Q14) and ‘Very stressful/Moderately stressful’ *vs*. ‘It does not upset me at all’ (Q32).

### Statistical analysis

Distribution of answers subdivided into the respective groups were compared with Chi-square test or Fisher’s exact test, where appropriate. P-value of 0.05 was considered statistically significant. Data was processed in Microsoft Excel 2017 (Microsoft, Washington, USA) while statistical analysis was performed using MedCalc statistical software, version 19.5.2 (MedCalc, Ostend, Belgium).

## Results

A total of 225 out of 297 employees responded to the survey, yielding an overall response rate of 76%, of which 118 were from the University Hospital Center Zagreb, 63 from UH Merkur and 44 from UH Dubrava, with individual response rates of 62%, 98% and 100%, respectively. Detailed participants’ demographic data, as revealed by the first part of the survey, are listed in Table 1.

**Table 1:** Participants’ general characteristics

**Question**	**Answers (N = 225)**	**N (%)**
1. Gender	Female	206 (92)
	Male	19 (8)
2. Age	≤ 40	97 (43)
	> 40	128 (57)
3. Work experience (years)	≤ 10	67 (30)
	> 10	158 (70)
4. Work experience at the current workplace (years)	≤ 10	92 (41)
	> 10	133 (59)
5. Level of education	Medical laboratory technician (graduated from a 4-year secondary school)	94 (42)
	Bachelor of Medical Laboratory diagnostics (a 3-year university degree)	59 (26)
	Master degree (in medical biochemistry, biology or medicine)	29 (13)
	PhD and/or specialist in Laboratory Medicine	43 (19)
6. Laboratory workplace	Preanalytical section	26 (12)
	Emergency laboratory	48 (21)
	Core laboratory	64 (28)
	Specialized laboratory	82 (36)
	No answer	5 (2)
7. In general, how satisfied are you with your workplace and work environment?	Very satisfied	63 (28)
	Moderately satisfied	129 (57)
	Moderately unsatisfied	23 (10)
	Very unsatisfied	10 (4)
8. Have you ever worked in a non-accredited laboratory?	Yes	166 (74)
	No	59 (26)
9. What is your level of responsibility regarding accreditation documents?	Evidence of work processes in predefined forms (evidence of routine activities regarding all phases of the TTP including environmental conditions, laboratory equipment, reagents, non-conformities *etc*.)	158 (70)
	Preparation of accreditation documents	54 (24)
	Approval of accreditation documents	12 (5)
	No answer	1 (0.4)
TTP - total testing process; PhD – Doctor of Philosophy.		

Participants’ general attitude towards accreditation and familiarity with ISO 15189:2012 (Q10-Q12) are presented in Table 2. Table 3 presents the distribution of answers concerning specific issues regarding implementation of accreditation requirements into routine practice (Q13-Q30). Response rates regarding accreditation audits (Q31-Q34) are shown in Table 4.

**Table 2:** General attitude towards accreditation and familiarity with ISO 15189 requirements

**Question**	**Answers (N = 225)**	**N (%)**
10. If you could choose, in which laboratory would you prefer to work in:	Accredited	158 (70)
	Non-accredited	35 (16)
	I have never worked in a non-accredited laboratory, so I cannot answer	32 (14)
11. What is your general attitude towards laboratory accreditation?	Positive	127 (56)
	Negative	8 (4)
	Neutral	90 (40)
12. How familiar are you with the requirements of ISO 15189?	Completely	31 (14)
	Very well	73 (32)
	Moderately	85 (38)
	Not at all	36 (16)

**Table 3:** Specific issues about implementation of accreditation requirements in routine practice

**Question**	**Answers (N = 225)**	**N (%)**
13. Do you consider the requirements of accreditation / the quality system in your laboratory easily understandable and clearly explained?	I totally agree	95 (42)
	I partially agree	118 (52)
	I do not agree	9 (4)
	No answer	1 (0.4)
14. I am regularly and timely informed about new standard operating procedures and work instructions.	I totally agree	129 (57)
	I partially agree	84 (37)
	I do not agree	11 (5)
	No answer	1 (0.4)
15. My suggestions regarding accreditation and improvements within the quality system are taken into consideration:	Regularly	43 (19)
	Occasionally	116 (52)
	Rarely to never	65 (29)
	No answer	1 (0.4)
16. For the same number of patients and laboratory tests, work in an accredited laboratory:	Increases the usual workload	152 (68)
	Does not affect the usual workload	60 (27)
	Decreases the usual workload	13 (6)
17. What do you consider to be the main advantages of accreditation?	Greater reliability of results	56 (25)
	Better documentation of the TTP	102 (45)
	Better understanding of analyses (including interferences, sample stability, *etc*.)	28 (12)
	There are no advantages	20 (9)
	Other: All answers are applicable (3/5) Comparability with other accredited laboratories (1/5) Traceability of the laboratory workflow (1/5)	5 (2)
	No answer	14 (6)
18. What do you consider to be the main disadvantages of accreditation?	Excessive workload	19 (8)
	Excessive paperwork	140 (62)
	Partial implementation of defined working procedures	24 (11)
	There are no disadvantages	16 (7)
	Other: Questionable applicability of accreditation requirements and defined working procedures (2/3) All answers are applicable (1/3)	3 (1)
	No answer	23 (10)
19. Do you follow the defined protocols during routine work?	Completely	201 (89)
	Partly	23 (10)
	Not at all	0 (0)
	No answer	1 (0.4)
20. Which proportion of your working time pertains to accreditation obligations?	< 25%	96 (43)
	26-50%	95 (42)
	51-75%	21 (9)
	> 75%	11 (5)
	No answer	2 (1)
**Question**	**Answers (N = 225)**	**N (%)**
21. In my opinion, compared to the period before accreditation, the results of the laboratory analyses are:	More reliable	79 (35)
	Equally reliable	106 (47)
	Less reliable	4 (2)
	I have not previously worked in a non-accredited laboratory	34 (15)
	No answer	2 (1)
22. Do you consider management of accreditation documentation in your laboratory:	Simple	7 (3)
	Appropriate	153 (68)
	Complicated	64 (28)
	No answer	1 (0.4)
23. TAT evaluation and monitoring:	Improves the workflow and reduces the time required to deliver laboratory test results	60 (27)
	Causes additional stress with no change in time required to deliver laboratory test results	100 (44)
	Does not affect the workflow	41 (18)
	I have no opinion	23 (10)
	No answer	1 (0.4)
24. I consider keeping regular evidence of temperature of the laboratory working space and refrigerators useful:	Yes	169 (75)
	No	55 (24)
	No answer	1 (0.4)
25. Evidence of non-conformities:	Points out to the drawbacks and improves the workflow	144 (64)
	Further slows down the workflow without affecting its quality and effectiveness	40 (18)
	I have no opinion	39 (17)
	No answer	2 (1)
26. Waste segregation and disposal:	Requires too much paperwork	21 (9)
	Is simple and environmentally acceptable	163 (72)
	I do not have an opinion	39 (17)
	No answer	2 (1)
27. Standard operating procedures and work instructions:	Are useful in routine work	160 (71)
	Are sometimes useful in routine work	31 (14)
	Are demanding to create and insufficiently used	28 (12)
	I do not have an opinion	3 (1)
	No answer	2 (1)
28. In my opinion, keeping evidence about all laboratory activities using predefined forms (temperature evidence, analyzer maintenance, critical values notification, *etc.*):	Contributes to better traceability of the laboratory workflow	159 (71)
	Increases the possibility of errors due to increased workload	29 (13)
	I do not have an opinion	35 (16)
	No answer	2 (1)
29. Regular assessment of staff competence for routine work:	Increases my work efficiency and reduces the possibility of mistakes	92 (41)
	Does not affect my working practice	87 (39)
	Increases the paperwork and causes additional stress without increasing my work efficiency	45 (20)
	No answer	1 (0.4)
**Question**	**Answers (N = 225)**	**N (%)**
30. With the introduction of accreditation, more attention is being given to employees’ continuing professional development:	I completely agree	60 (27)
	I partially agree	112 (50)
	There is no change	52 (23)
	No answer	1 (0.4)
TTP - total testing process. TAT – turnaround time.		

**Table 4:** Accreditation audits

**Question**	**Answers (N = 225)**	**N (%)**
31. The dynamics of periodical internal audits is:	Too frequent	30 (13)
	Adequate	185 (82)
	Not frequent enough	10 (5)
32. How stressful do you experience periodical internal audits?	Very stressful	16 (7)
	Moderately stressful	127 (57)
	It does not upset me at all	82 (36)
33. How stressful do you experience the annual accreditation audit?	Very stressful	54 (24)
	Moderately stressful	125 (56)
	It does not upset me at all	46 (20)
34. How would you prefer the next accreditation audit to be conducted?	On-site	55 (24)
	Online (virtual)	164 (73)
	No answer	6 (3)

Evaluation of comparisons between the selected subgroups showed that there are statistically significant differences for the largest number of questions in the comparison between technical and academic staff. Specifically, more technicians revealed a neutral attitude towards accreditation (75/153 *vs.* 15/72; P<0.001), consider themselves not at all (34/153 *vs*. 2/72) or only moderately familiar (71/153 *vs*. 14/72) with ISO 15189:2012 (P < 0.001), partly agree that they are regularly informed about new operating procedures (62/153 *vs.* 22/72; P = 0.048) or that their suggestions regarding accreditation are taken into consideration regularly (P < 0.001), and are not predominantly aware about the value of monitoring non-conformities (P = 0.012).

Regarding turnaround time (TAT) evaluation and monitoring, more technicians and more employees from the routine laboratory think that it introduces additional stress without any change in time required to deliver laboratory test results (P = 0.008 and P < 0.001, respectively).

Additionally, evaluation of the impact of accreditation on the workload revealed a statistically significant difference by work experience (P = 0.022), duration of work at the same workplace (P = 0.020) and level of education (P = 0.020), with more employees with longer work experience as well as academic staff considering that accreditation has increased the usual workload.

The question about regular evidence of laboratory activities via structured forms yielded a statistically significant difference by work experience (P = 0.011) and duration of work at the current workplace (P = 0.007), with more employees with longer experience revealing no opinion.

Comparison between answers from participants divided per age yielded no statistical difference for any of the questions.

Distribution of answers between the assessed laboratories revealed some significant differences, as presented in Table 5. Further evaluation of the distribution of answers between the one laboratory accredited for 14 years, compared to the two others accredited for 7 years, revealed an evident overall greater rate of positive responses among employees from UH Merkur compared to UHC Zagreb and UH Dubrava, with only a few questions not yielding statistically significant difference, *i.e.* questions 15 (P = 0.194), 20 (P = 0.941), 27 (P = 0.533), 31 (P = 0.325), 32 (P = 0.689) and 33 (P = 0.612).

**Table 5:** Response frequencies of one relevant answer for questions where statistically significant difference was observed between the assessed laboratories

	**UHC Zagreb,** **N (proportion)**	**UH Merkur,** **N (proportion)**	**UH Dubrava,** **N (proportion)**	**P**
Q11: Positive attitude towards accreditation	64 (0.54)	47 (0.75)	16 (0.36)	< 0.001
Q12: Complete / very good familiarity with the requirements of ISO 15189	46 (0.39)	38 (0.60)	20 (0.45)	0.023
Q13: Agree that the requirements of accreditation / the quality system are easily understandable and clearly explained	43 (0.36)	36 (0.57)	16 (0.36)	0.025
Q14: Agree that they are regularly and timely informed about new standard operating procedures and work instructions	63 (0.53)	46 (0.73)	20 (0.46)	0.009
Q16: Accreditation increases the usual workload	79 (0.67)	33 (0.52)	40 (0.91)	< 0.001
Q21: Accreditation increases the reliability of laboratory results	34 (0.29)	27 (0.43)	18 (0.41)	0.034
Q23: TAT monitoring improves the workflow and reduces the time required to deliver laboratory test results	30 (0.13)	23 (0.37)	7 (0.16)	0.003
Q25: Evidence of non-conformities points out to the drawbacks and improves the workflow	63 (0.53)	53 (0.84)	28 (0.64)	< 0.001
Results are presented as absolute numbers and proportions of the total number of survey participants *per* each laboratory (UHC Zagreb N = 118, UH Merkur N = 63 and UH Dubrava N = 44). P<0.05 was considered statistically significant. Q – question. UHC - University Hospital Center. UH - University Hospital. TAT - turnaround time.				

## Discussion

The presented results of the first survey on attitudes about accreditation conducted among employees of three Croatian medical laboratories accredited in compliance with ISO 15189:2012 demonstrate that there is an overall positive attitude towards accreditation and an existing awareness of the benefits it offers, supporting the results from previously published surveys (*9*, *14*, *15*). However, several specific issues have been revealed through this survey that clearly deserve further attention and consideration.

This survey unequivocally shows that laboratory staff recognizes the value of the organized wokflow imposed by the accreditation standard, being especially pleased with the availability of written procedures, regular evidence of all laboratory processes via forms and the identification of non-conformities, which are considered valuable contributors for efficient traceability of the TTP. On the contrary, increased workload, caused by excessive paperwork that requires substantial time commitment, is recognized as the main disadvantage of accreditation, as equally observed in other laboratory settings (*9*, *14*, *16*). Further concerns that arised from the survey, and which were more strikingly reported by technical staff, include lack of familiarity with ISO 15189:2012, unclear explanations of accreditation requirements, inadequate informing about new operating procedures and working instructions within their workplace as well as only occasional acceptance of their suggestions about improvements of the quality system. These findings call for a shift in the existing mind-set that the quality system needs to be envisioned and organized exclusively at the academic level, but also emphasize that there is an urgent need for introducing mechanisms for accurate and timely downstream information flow. Only in this way, technical staff can fully comply with accreditation requirements and working protocols. Given that they follow prescribed protocols daily, their first-line experience and suggestions should serve as valuable inputs for improvements of the TTP, thus proving the actual appreciation of their competence as well as being highly encouraging for the individual (*17*, *18*).

Moreover, our survey shows that laboratory staff is not completely satisfied with the access to continual professional development programmes and does not consider the currently used formats for regular competence assessment as effective means for improving laboratory staff working performance. Indeed, the predominantly used method for competence assessment in the surveyed laboratories is through written exams, which is from earlier known as a method of evaluation characterized by the poorest compliance and therefore not recommended unless combined with practical tasks (*19*). In accordance with this, existing practices should be obviously modified and replaced with more attractive and effective formats of competence assessment, preferably real life practical tasks which should be tailored to the requirements of the specific laboratory area (*19*).

Regular assessment of implementation and effectiveness of the defined processes and quality management system is another important feature required for the success of accreditation and must be performed regularly through internal audits divided by sections, with an entire cycle completed within one year (*20*, *21*). Our survey participants encourage the bimonthly dynamics of internal audits in their laboratories, being experienced as stressful only occasionally, while annual on-site surveillance visits conducted by the national accreditation body are being considered prominently more stressful.

In the light of continual improvement, ISO 15189:2012 promotes the establishment of quality indicators as measurable, quantitative and objective tools in order to ensure periodical, systematic monitoring of processes and identifying areas which might need improvement. Turnaround time is the most widely applied quality indicator, introduced with the main purpose to enhance quality of laboratory services by timely delivery of laboratory results which in turn might closely follow clinical needs (*21*-*23*). However, we evidenced that such monitoring caused a rather unexpected counter effect among technical staff, especially within the STAT and core laboratory, being considered only as an additional stressor. It can be speculated that this is due to a significantly shorter TAT assigned to the tests performed, compared to specialized laboratories. Turnaround time in the emergency laboratory setting is set at 60 minutes, which inevitably causes mounting pressure among staff when preanalytical or analytical processes get delayed for any number of reasons. The results obtained herein serve as a reminder that analytical quality should never be sacrificed for a faster TAT and that our efforts should not be focused on obsessively measuring TAT but rather on identifying bottlenecks and introducing improvements within the laboratory workflow (*23*).

This study also evidenced a greater positive attitude and better compliance with ISO 15189:2012 requirements among employees from the laboratory that has been accredited for the longest period (14 *vs.* 7 years). This not only points out to the fact that individual ways of fulfilling the ISO 15189:2012 requirements may vary among institutions and elicit different reactions and attitudes, but more importantly, confirms results from a previous study that over the course of time people become accustomed and better adopt accreditation requirements (*9*).

The main limitation of this study pertains to all surveys – the answers were anonymus, free and subjective, thus participants might not have provided true information and this could have possibly introduced bias in the final results. The evaluation of staff attitudes just one week before the annual accreditation visit is another possible source of bias due to the widely declared increased stress caused by the upcoming audit, which was at the time additionally complicated by COVID-19 restrictions and made uncertain whether the visit would be conducted on-site or online. Also, the survey included only 3 out of 8 accredited medical laboratories in Croatia; however, all of them are fairly large laboratories belonging to prominent clinical hospitals and thus encompass a representative number of employees from Croatian accredited laboratories. In addition, this survey offers a general overview of laboratory professionals’ attitudes towards accreditation. Thus, further surveys focused on specific areas of accreditation not included in detail herein are required. To the best of our knowledge, no validated guidelines for survey design are available and recommended quality criteria for survey research and reporting vary significantly, therefore, our survey design was based on similar published questionnaires, which could have affected its overall quality (*24*). Nevertheless, we believe that, as a first survey of this type, *i.e.* dealing with staff satisfaction concerning accreditation of medical laboratories in Croatia, it gives an objective and valuable insight into implementation of accreditation into routine practice, reveals its strengths and weaknesses, and can serve as a starting point for re-evaluation of current practices and introduction of possible improvements.

In conclusion, medical laboratory staff within the assessed clinical hospitals seem to be well-aware of the value of accreditation and are mostly satisfied with the way it is implemented in routine practice, despite the stated additional workload. Improvements are needed in providing better education for technical staff about ISO 15189:2012, in order to better understand the rationale behind its requirements, as well as the impact of accreditation on the quality of laboratory results. There is a clear need for introducing more efficient and user-friendly ways of competence assessment. Another burning issue is improving access to continuing professional development which is recognized as a strong contributor to job satisfaction. Successful implementation of ISO 15189:2012 can be achieved only through ongoing active involvement and proven competence of the entire laboratory staff. Appropriate training ensures full conformity with established protocols and activities, regardless of the level of education and responsibility. In this manner, each individual employee might effectively contribute to the continuous improvement of laboratory services (*2*).
